# Protein Intake and Muscle Health in Old Age: From Biological Plausibility to Clinical Evidence

**DOI:** 10.3390/nu8050295

**Published:** 2016-05-14

**Authors:** Francesco Landi, Riccardo Calvani, Matteo Tosato, Anna Maria Martone, Elena Ortolani, Giulia Savera, Emanuela D’Angelo, Alex Sisto, Emanuele Marzetti

**Affiliations:** Department of Geriatrics, Neurosciences and Orthopedics, Catholic University of the Sacred Heart, L.go F. Vito 8, Rome 00168, Italy; riccardo.calvani@gmail.com (R.C.); matteo.tosato@rm.unicatt.it (M.T.); annamariamartone@gmail.com (A.M.M.); eleort@gmail.com (E.O.); giulia.savera@libero.it (G.S.); manud1983@yahoo.it (E.D.); alexsisto@gmail.com (A.S.); emarzetti@live.com (E.M.)

**Keywords:** sarcopenia, frailty, malnutrition, leucine, anorexia, supplementation, amino acid

## Abstract

The provision of sufficient amounts of dietary proteins is central to muscle health as it ensures the supply of essential amino acids and stimulates protein synthesis. Older persons, in particular, are at high risk of insufficient protein ingestion. Furthermore, the current recommended dietary allowance for protein (0.8 g/kg/day) might be inadequate for maintaining muscle health in older adults, probably as a consequence of “anabolic resistance” in aged muscle. Older individuals therefore need to ingest a greater quantity of protein to maintain muscle function. The quality of protein ingested is also essential to promoting muscle health. Given the role of leucine as the master dietary regulator of muscle protein turnover, the ingestion of protein sources enriched with this essential amino acid, or its metabolite β-hydroxy β-methylbutyrate, is thought to offer the greatest benefit in terms of preservation of muscle mass and function in old age.

## 1. Introduction

**Figure 1:**
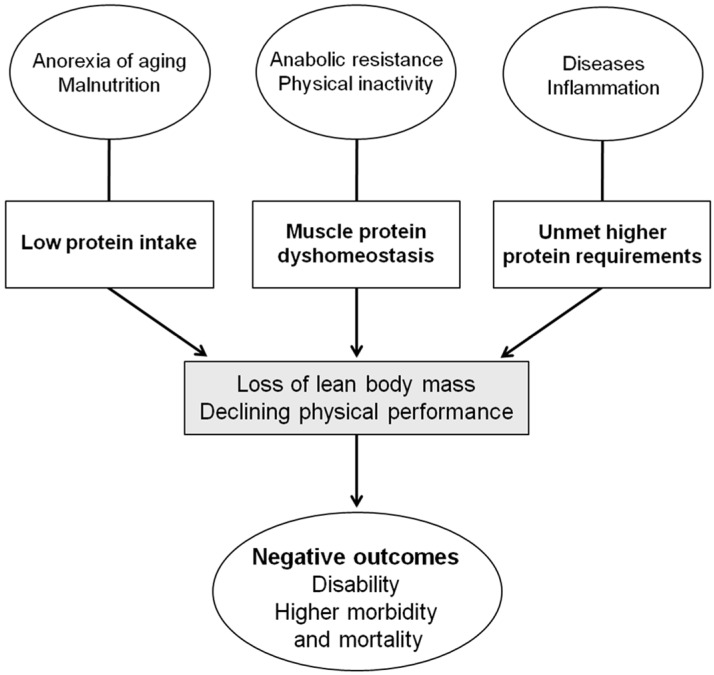
Alterations in protein homeostasis during aging and related clinical outcomes.

An adequate intake of dietary proteins is vital to maintaining muscle mass as it ensures the provision of essential amino acids and stimulates protein synthesis. Older individuals are at high risk for insufficient protein intake. Furthermore, the current recommended dietary allowance (RDA) for protein (0.8 g/kg/day) might not be sufficient for preserving muscle mass and quality in old age [1]. This, along with the fact that high protein ingestion decreases the risk of perioperative complications [2,3], increases bone mineral density [4,5], and reduces rehabilitation time after an acute disease [6], highlights the importance of optimal protein ingestion in later life. However, it is debated whether the current RDA for protein intake is truly sufficient to avoid major adverse events in older persons, especially in frail, critically ill patients [7]. Given the fact that protein–energy malnutrition is a major risk factor for the development of sarcopenia [8,9,10], a suboptimal nutritional status may mediate the association between sarcopenia and negative clinical outcomes in older and frail individuals (Figure 1).

For example, in the Health, Aging, and Body Composition (Health ABC) Study, older participants in the highest quintile of protein intake lost nearly 40% less appendicular lean mass than did those in the lowest quintile over three years of follow-up, after adjusting for potential confounders [11]. In this respect, it is important to highlight that protein supplementation *per se* may be sufficient to increase muscle mass in malnourished older inpatients [12]. This effect may clarify, at least partially, the improvement in clinical outcomes observed in older hospitalized persons undergoing specific protein supplementation regimens [7,13]. This evidence emphasizes the central role of nutrition and in particular, the correct level of protein consumption in the management of sarcopenia.

One crucial aspect to consider is that a greater dietary protein intake may be necessary for the promotion of muscle health in older individuals compared with younger persons [7]. Yet, a number of issues related to protein intake and eventually protein supplementation in old age must still be addressed.

## 2. Putative Mechanisms of Protein Action on Muscle Cells

Skeletal muscle mass is controlled by the complex interaction of many factors; nevertheless, it is indisputable that the balance between protein synthesis and breakdown has an integral role in the process [14]. Significant losses or improvements in muscle mass are therefore primarily the result of persistent changes in muscle protein synthesis rates, breakdown rates, or a combination of both mechanisms. On the other hand, muscle protein metabolism is greatly dependent upon ingesting an adequate amount of proteins and amino acids [15,16].

Muscle protein synthesis rates are controlled predominantly by responsiveness to anabolic stimuli, such as physical activity and food consumption [17]. Dietary protein and/or specific amino acid ingestion largely increases muscle protein synthesis rates and inhibits protein breakdown, thus favoring net muscle protein deposition. In particular, aging does not seem to influence skeletal muscle protein breakdown, autophagy, or the ubiquitin proteasome system, for example following an acute bout of resistance exercise [18]. Therefore, targeting the muscle protein synthesis response may hold more promise in the prevention sarcopenia during the aging process.

The amino acid content of dietary proteins has a significant impact on their anabolic power [19]. In fact, essential amino acids (EAAs) are the main nutritional stimulus for protein synthesis. Leucine is considered the principal dietary regulator of muscle protein anabolism [20], due to its capability to activate the mammalian target of rapamycin (mTOR) pathway and inhibit the proteasome [8]. Even though during the aging process muscle shows a decreased anabolic response to low doses (e.g., 7 g) of EAAs [8], higher doses (e.g., 10–15 g, with at least 3 g of leucine) are able to overcome these anabolic resistances and stimulate a protein synthetic response similar to that mounted by younger adults [8]. Consequently, older individuals should be advised to consume protein sources containing high proportions of EAAs (*i.e.*, high-quality proteins), such as lean meat and dairy-based products, and other leucine-rich foods (e.g., soybeans, peanuts, cowpea, lentils). It is important to highlight that plant-based sources are likely to result in a lower muscle anabolic response upon ingestion when compared with animal-based proteins [21]. In particular, the ingestion of soy protein results in a reduced muscle anabolic response in older individuals relative to the ingestion of whey [22] or beef [23], most likely due to a lower leucine content and/or a lack in specific EEAs, such as methionine and/or lysine.

Anabolic resistance reflects the inability of skeletal muscle to maintain protein mass through the appropriate stimulation of protein synthesis. Recently it has been demonstrated that fast-digesting soluble milk protein improves postprandial muscle protein synthesis especially within the mitochondrial compartment and the myosin fractional synthesis rate, in older persons [24]. At the same time, it should be emphasized that decreased levels of physical activity play a major role in determining muscle anabolic resistance [25]. Indeed, physical inactivity and muscle disuse lead to the rapid development of anabolic resistance in both young and older individuals [26,27,28]. Reduced physical activity is also expected to lower basal muscle protein synthesis rates. Finally, several other lifestyle and biological factors, such as smoking, alcohol abuse, hormonal status, various disease conditions, and chronic inflammation may strongly influence muscle protein metabolism [29,30,31].

Insulin plays a particularly important role in protein metabolism [32]. Amino acids and insulin have a synergistic effect on stimulating muscle protein anabolism [33]. In physiological conditions, maximum protein anabolism occurs throughout the fed state, during which concentrations of insulin and amino acids are higher. In particular, the postprandial increase in insulin to stimulate protein synthesis and to inhibit breakdown is permissive rather than modulatory [34]. The postprandial rise in amino acid concentrations dictates the muscle anabolic response.

## 3. Dietary Protein Requirements: How Much Protein Is Enough for Older Adults?

A dietary protein requirement is defined as the amount of protein or its constituent amino acids, or both, that must be supplied in one’s diet in order to satisfy metabolic demand and achieve nitrogen equilibrium. For more vulnerable people, like pregnant or lactating women, older and diseased persons, newborns and children, the provision of adequate dietary protein intake is crucial for growth and/or the preservation of muscular homeostasis.

Skeletal muscle mass and functional capacity are controlled by the dynamic interaction of numerous factors, also encompassing diet and nutrition. An adverse yet typical consequence of the aging process is the progressive loss of muscle mass and physical function, named sarcopenia. Although the onset and progression of sarcopenia can be influenced by many factors, a compromised capacity to maintain the anabolic response after dietary protein intake [35] has become a key target for researchers.

Independent of the type of protein and its source, it is important to underline that meals should include an appropriate amount of high-quality protein. In recent years, consensus statements and opinion articles have asserted that protein intake above 0.8 g/kg/day may confer muscle health benefits greater than those conferred by the current RDA [36,37,38,39].

As such, a protein intake of 1.0–1.2 g/kg/day has been recommended for the preservation of healthy aging muscles, while 1.2–1.5 g/kg/day of protein may be necessary in older patients with acute or chronic diseases [7,14,33]. Elderly people with severe illness or malnutrition may need as much as 2.0 g/kg/day of protein [7].

Epidemiological studies have suggested that low protein intake is correlated with the onset and progression of sarcopenia. Recently, it has been demonstrated that a low intake of calories, protein, and leucine is associated with reduced muscle mass in hip-fractured elderly [40]. Given the relevance of sarcopenia as a risk factor for adverse outcomes in frail older persons [41,42], these findings emphasize the importance of a comprehensive dietary assessment for the detection of nutritional deficits that predispose to or aggravate muscle atrophy.

Tieland and colleagues [43,44] reported the findings of two trials on the effect of protein supplementation on muscle mass and physical performance in frail older participants. They determined that a 24-week supplementation of a beverage containing 15 g milk protein concentrate administered twice-daily improved muscle strength and physical performance, but did not increase muscle mass. A similar supplementation regimen increased muscle mass when combined with resistance exercise training. Interestingly, in both trials, after including the dietary supplements, the daily protein intake in the intervention group increased up to 1.4 g/kg/day. However, it is important to highlight that it is likely that the older persons who had greater protein intakes and displayed greater strength and muscle size also had higher intakes of other nutrients, which may also explain their improved status. Nevertheless, protein metabolism is dynamic in nature and can become more efficient under specific conditions such as exercise training and a well-balanced diet.

## 4. Protein Quality: Which Is the Best Protein Source?

Besides quantity, the quality of consumed protein has a critical role in the context of muscle health [8,45,46]. Amino acids have been traditionally categorized as indispensable (essential), conditionally indispensable, or dispensable (nonessential), based on the capacity of the body to synthesize the amino acid from other carbon sources. Dietary protein sources are often assorted in their amino acid content; however, differences in EAA profile, digestibility, and bioavailability determine the anabolic properties (or quality) of specific protein sources [47]. Ingesting EAAs, which cannot be synthesized *de novo* and have primary role in the regulation of muscle protein synthesis, can potentially mitgate the loss of muscle protein during the aging process. At the same time, the ingestion of EAAs provides a more efficient nutritional approach (*i.e.*, greater stimulation of protein synthesis relative to the amount of amino acids ingested) to enhance muscle protein synthesis compared with the ingestion of intact protein.

### 4.1. Fast versus Slow Proteins

The absorption kinetics and the amino acid composition of dietary proteins are important factors that must be considered. The rapidity of absorption of dietary amino acids by the gut influences the postprandial protein synthesis rate, breakdown, and, finally, protein deposition [48]. This observation has contributed to the development of the fast *versus* slow protein model [48], which may have significant implications for the prevention and treatment of sarcopenia. In young individuals, slowly digested proteins (e.g., casein) may produce greater protein retention than those that are more quickly digested (e.g., whey). An opposite pattern has been documented in older individuals [49]. Accordingly, some authors have demonstrated that the intake of whey protein stimulates postprandial muscle protein deposition in older men more efficiently than casein or casein hydrolysate [50]. Consequently, the concept of “fast” *versus* “slow” protein may need to be considered when developing specific nutritional recommendations as well as the prescription of specific supplements.

### 4.2. Animal- versus Plant-Derived Proteins

No definitive evidence is available regarding the possibly differing effects of animal-derived and plant-based proteins on muscle health in old age. Few studies have compared the capacity of animal- *versus* plant-derived proteins to influence muscle mass or function [51]. Plant-based proteins generally contain smaller quantities of EAAs and are less digestible than animal-derived proteins. This is predominantly due to lower amounts of lysine, methionine, and/or leucine in plant-based proteins. Hence, it is expected that the ingestion of greater amounts of plant-based proteins per meal may be necessary to achieve the same anabolic response evoked by smaller quantities of animal-derived proteins [21]. However, recent findings in elderly men indicate that the consumption of large amounts of plant-based proteins, as a strategy to ameliorate muscle protein synthesis, may not be as effective as expected [22]. Indeed, the authors showed that amino acids derived from soy protein were directed more toward oxidation than used for *de novo* muscle protein synthesis compared with whey protein-derived amino acids.

Meat is an excellent source of high quality proteins, which are essential for optimal muscle and bone development. Meat contains a large quantity of EAAs and it is important to highlight that the intake of EAAs from meat, for the same weight, is higher than all other foods. The correct intake of biologically active compounds contained in meat, such as creatine, carnitine, and other nutrients such as iron and cobalamin, have a significant impact upon human protein metabolism and so likely have beneficial effects on the prevention of sarcopenia [52]. In this respect, within a varied and balanced diet aimed at preventing sarcopenia, consuming meat 4–5 times a week (*i.e.*, white meat two times per week, lean red meat less than two times per week, and processed meat less than two time per week) should be recommended.

### 4.3. Physical Properties of Protein Sources

The formulation of protein sources has an important effect on protein digestion, absorption, as well as whole-body and muscle anabolism. The ingestion of amino acids in liquid form produces a greater postprandial amino acid concentration compared with the same amino acids consumed in solid form [51]. However, some studies demonstrated that, even though minced beef was more rapidly digested and absorbed than whole steak, the difference did not translate into greater postprandial muscle protein synthesis rates [53]. Further studies are necessary to assess the significance of not only the “biological” quality but also the “physical” quality of protein sources and the specific milieu in which the protein is eaten.

## 5. Protein Ingestion Timing: When Is It Best to Consume Protein?

The timing of protein ingestion and the synergistic effect of protein intake with physical activity are relevant factors to consider in enhancing muscle health.

### 5.1. Protein Feeding Patterns

The most significant issue regarding the timing of protein intake is the pattern of ingestion. Arnal and colleagues [54] documented that, in older persons, a two-week protein pulse-feeding pattern in which 80% of daily protein intake was delivered in one meal was more effective in enhancing whole-body protein retention than the same amount of protein distributed uniformly across four meals. However, ingesting large quantities of proteins in a single meal may be difficult to maintain over the long term, particularly in old age. Moreover, most researchers agree proteins should be introduced uniformly throughout the day in order to guarantee a more sustained 24-h anabolic response [35,39]. Hence, older persons should be encouraged to eat between 1.0 and 1.2 g/kg of body weight per day of protein through the consumption of an adequate serving (e.g., 25–35 g) of high-quality protein sources at every meal [35,39]. The resulting spread-feeding pattern, in which a similar quantity of proteins is consumed at every meal, would ensure a 24-h stimulus of protein synthesis [54].

The protein meal threshold theory has been addressed by recent findings on the differential effects of 10, 20, and 35 g of whey protein during a primed continuous intravenous infusion of phenylalanine and tyrosine (l-[ring-2H5]phenylalanine and l-[ring-2H2]tyrosine) in healthy older men [55]. The authors demonstrated that amino acid absorption and the consequent stimulation of muscle protein synthesis were suboptimal after the intake of 10 g of protein, slightly improved after a 20-g protein meal and were greatest and statistically significant only after the ingestion of 35 g [55]. Other recent studies have shown the possible importance of meal distribution patterns for optimal, repeated stimulation of muscle protein synthesis throughout the day [56,57]. On the other hand, other investigations examining the eating patterns of older adults support the idea that daily protein ingestion should be focused on individual meals or specific eating opportunities, with breakfast offering the best chance to more equally allocate protein throughout the day [57]. Considering the uncertainty of the current evidence, studies designed to specifically assess the effects of different patterns of protein ingestion are needed to develop nutritional interventions for the management of sarcopenia.

### 5.2. Protein Intake and Physical Exercise

A harmonized prescription of dietary protein and physical exercise may be important for middle-aged and older adults suffering from catabolic stressors such as illness, inflammation, or injury, or experiencing prolonged physical inactivity. The combination of physical exercise and protein intake has a positive and synergistic effect on skeletal muscle protein synthesis [58]. Some data suggest that resistance and aerobic exercise offers the greatest benefit to aged muscle when combined with a dietary protein intake that exceeds the current RDA [59]. Still, one of the major issues regarding protein intake is identifying when to ingest protein relative to physical exercise. The consumption of branched-chain amino acids combined with exercise training produces an anabolic environment [60]. Protein intake following physical exercise has positive effects on the balance between protein synthesis and breakdown, stimulating muscle protein anabolism [14].

Optimizing the timing of protein intake and exercise training is a “biologically plausible” approach to increase the potential for muscle protein anabolism. Some studies suggest that resistance exercise training momentarily constrains protein synthesis via increased AMP-activated protein kinase (AMPK) activation and decreased phosphorylation of eukaryotic translation initiation factor 4E-binding protein 1 (4E-BP1) and other specific key regulators of translation initiation [54]. However, 60 min after exercising, maximal muscle protein synthesis is restored and theoretically amplified via the activation of protein kinase B (Akt), mTOR, ribosomal protein S6 kinase beta-1 (S6K1), and eukaryotic elongation factor 2 (eEF2) [61,62]. Consequently, the highest level of muscle protein synthesis is observed around 60 min after the end of exercise training. Accordingly, improving the availability of amino acids throughout this period could offer the largest anabolic advantage [14]. However, even though protein synthesis in the first hour after exercise is maximized, the sensitivity of the muscle to protein feeding is enhanced for up to 24 h after exercise [63]. In this respect, it should be highlighted that repeated protein feeding in the post exercise period is important to optimally facilitate the muscle adaptive response.

For older persons, eating a moderate amount of protein (*i.e.*, 20–30 g/kg/day) at breakfast, lunch, and dinner could offer the required amino acid precursors and flexibility required to permit at least some protein–exercise anabolic synergy, regardless of when the exercise session or physical activities are performed.

## 6. Protein Supplementation: New Evidence

Robust evidence indicates that anorexia of aging is a major reason for overall and selective malnutrition [64]. Anorexia is associated with a higher risk of quantitative malnutrition (for example, protein-energy malnutrition) due to inadequate overall nutritional intake. However, in the early stages, anorexia increases the risk of qualitative malnutrition, due to the suboptimal intake of specific nutrients, like proteins and vitamins [65]. Studies have demonstrated that selective malnutrition is also related to the development of sarcopenia and several other negative health outcomes, including morbidity and mortality [10,66].

Nutritional supplementation does not directly treat anorexia of aging, only its consequences, such as weight loss and protein-energy malnutrition. A small number of studies have demonstrated positive effects from energy supplementation in malnourished older adults. Nevertheless, the heterogeneity of the supplementation protocols implemented impedes their applicability to standard patient care. As it stands, the only conclusive evidence is presently limited to protein supplementation. Recently, it has been demonstrated that β-hydroxy β-methylbutyrate (HMB)—an amino acid metabolite of leucine—is able to stimulate protein synthesis and improve muscle strength and body composition in older adults [67].

### 6.1. Nutritional Supplementation with Leucine

Given the role of leucine as the principal dietary regulator of muscle protein anabolism, supplementation with protein sources enriched with this EAA should provide substantial benefits in the preservation of muscle mass and function [35,68]. This view is in line with recent findings that both total protein ingestion and leucine intake are positively associated with muscle mass in hip-fractured elderly patients [40]. However, the effects of leucine supplementation in clinical studies have not been consistent. Differences in the results might be attributable to dissimilar study protocols, with some programs administering a large bolus of leucine over a short period of time and others serving a continuous infusion of leucine. Generally, the findings suggest that leucine supplementation has potential positive effects on muscle metabolism in older people and that there is a minimal dosage that produces these effects [40]. For example, one study assessed the effects of a single dose of branched-chain amino acids (6.7 g) with different amounts of leucine (2.8 *versus* 1.7 g) on postprandial protein synthesis in older persons, demonstrating that those supplemented with a higher dosage had a significant increase in protein synthesis compared with participants supplemented with a smaller dose of leucine [69]. More recently, the PROVIDE study demonstrated that a oral nutritional supplement containing vitamin D- and leucine-enriched whey protein improved in muscle mass and lower extremity function in sarcopenic older adults [70]. The active group achieved a higher total protein intake (1.5 g/kg/day) during the study period, which is in line with the recent PROT-AGE recommendations for older populations [7]. Finally, leucine supplementation has been shown to enhance the muscle anabolic response when suboptimal amounts of protein are consumed [71,72,73].

### 6.2. Nutritional Supplementation with HMB

HMB is an active leucine metabolite which activates the mTOR signaling pathway in muscle. Following its absorption, dietary leucine is converted into α-ketoisocaproate (KIC), which is further metabolized into either isovaleryl-CoA or HMB. Under normal conditions, the majority of KIC is converted into isovaleryl-CoA, while only approximately 5% of leucine is metabolized to HMB. This implies that, in order to reach pharmacological levels of HMB, this compound needs to be administered directly, rather than via increasing leucine dosage. It has recently been suggested that HMB may be used to protect or restore muscle mass in older persons with reduced lean body mass [74].

HMB exerts its effects through protective, anticatabolic mechanisms and directly influences protein synthesis. HMB has also been shown to stabilize the muscle cell membrane, to modulate protein degradation and to up-regulate protein synthesis [68]. The daily administration of a nutritional mixture including HMB (2 g), arginine (5 g), and lysine (1.5 g) for 12 weeks was shown to improve physical performance, muscle strength, fat-free mass and protein synthesis in sedentary older women [74]. More recently, Deutz and colleagues [13]—in a multicenter, randomized, placebo-controlled, double-blind trial—demonstrated that the early administration (within 72 h of hospitalization) of a nutrient-dense oral nutritional supplement containing high concentrations of protein and HMB was associated with decreased post-discharge mortality and improved nutritional status in malnourished older adults [13].

Similar to leucine, HMB holds promise when suboptimal amounts of protein are consumed. In short, HMB has emerged as a promising candidate for nutritional interventions against sarcopenia, but more extensive studies are needed to establish the optimal dosage and possible side effects resulting from chronic supplementation.

## 7. Conclusions

The recognition of sarcopenia as a major risk factor for adverse outcomes in frail older populations indicates that skeletal muscle may represent a critical target for interventions. The association between low energy and protein intake and reduced muscle mass in older adults, revealed by several studies [40,64,75], highlights the importance of a comprehensive dietary assessment for the early detection of nutritional deficits, which may promote muscle loss. The suboptimal consumption of protein is often the case for older individuals. When nutritional supplements are suggested as a strategy to improve muscle health, providing protein (containing a variety of amino acids) may be preferred over the ingestion of a single amino acid (such as leucine, let alone its metabolite HMB). Given the heterogeneity of the older population, it is likely that, as opposed to standardized nutritional regimens, the greatest health benefits may be achieved through the provision of personalized dietary recommendations [76]. Finally, in older persons engaged in physical activity, nutritional interventions should be structured to take advantage of the anabolic window “opened” by physical exercise.
